# Improved DC Dielectric Performance of Cross-Linked Polyethylene Modified by Free Radical-Initiated Grafting BMIE

**DOI:** 10.3390/ma16206659

**Published:** 2023-10-12

**Authors:** Peng Li, Xuan Wang, Jin Jin, Hui Zhang, Wei Han

**Affiliations:** Key Laboratory of Engineering Dielectrics and Its Application of Ministry of Education, Harbin University of Science and Technology, Harbin 150080, China; lp_hrbust@126.com (P.L.); emailatjin@163.com (J.J.); huizhang@hrbust.edu.cn (H.Z.); charles_han@fjnu.edu.cn (W.H.)

**Keywords:** cross-linked polyethylene, bis(maleimido)ethane, space charge, carrier trap, free radicals

## Abstract

To enhance the direct current (DC) dielectric properties of cross-linked polyethylene (XLPE) for high-voltage (HV) cable insulation, the polyethylene molecular chain is modified by grafting bismaleimide ethane (BMIE), which creates carrier deep traps within the polymer material. Compared to the traditional modified molecule maleic anhydride (MAH), BMIE has a significantly higher boiling point than the production temperature of XLPE. Additionally, it does not release bubbles during the production process and, thus, preserves the dielectric properties. It was proved by infrared spectroscopy and a gel content test that BMIE was successfully grafted onto the polyethylene molecular chain and had no effect on the crosslinking degree of the polymer while reducing the amount of crosslinker, thereby reducing the influence of the by-products of the decomposition of dicumene peroxide (DCP) on the electrical resistance of polymers. The analysis of DC breakdown field strength, current density, and space charge distribution at various temperatures demonstrates that grafting BMIE can greatly enhance the dielectric properties of insulation. Polar groups in the BMIE molecule create deep trap energy levels in XLPE-g-BMIE, and these trap energy levels contribute to the formation of a charged layer near the electrode, which is shielded by Coulomb potential. As a result, the charge injection barrier increases. Additionally, the presence of these polar groups reduces the mobility of charge carriers through trap-carrier scattering, effectively suppressing the accumulation of space charge within the material. First-principle calculations also confirm that bound states can be introduced as carrier traps by grafting BMIE onto polyethylene molecules. The agreement between experimental results and simulation calculations indicates that grafting BMIE to enhance the dielectric properties of polyethylene is a new and feasible research direction in the exploitation of materials for HVDC cables.

## 1. Introduction

With the high-speed growth of human society and the strengthening of environmental awareness, clean and renewable power generation, i.e., green power, has become the focus of research and development for the future development of power systems, and its power generation accounts for an increasingly high proportion [1]. At present, several major new energy generation methods, such as wind, solar, and other power systems, have the characteristics of reverse distribution and wide distribution with the power load center, so how to solve the problem of acceptance of the above clean energy in the transmission network has become an important factor influence on the development of the power system. With the development of electronic and electrical technology, high-voltage DC transmission has been able to achieve long-distance, high-capacity, low-loss power transmission; therefore, high-voltage DC transmission technology has become the main choice of new energy transmission methods [2]. As an important part of the HVDC transmission, the insulation performance of the cable is subject to higher requirements to guarantee the safety and reliability of the transmission system [3,4,5]. Cross-linked polyethylene (XLPE) has been the dominant insulation material in DC cables since the 1960s because of its outstanding thermal and electrical properties and cost advantages. XLPE maintains the characteristics of high insulation resistance, high dielectric breakdown strength, low dielectric constant and dielectric loss of polyethylene (PE), and also has better thermal and mechanical properties than PE. But, as XLPE is used as cable insulation on a huge scale, many issues and technical problems in the production, operation, and recycling of XLPE have appeared progressively, under the action of HVDC electric field, XLPE is prone to charge injection and space charge accumulation, leading to electric field distortion, insulation aging, and even dielectric breakdown. In addition, XLPE cable manufacturing mainly uses DCP as a crosslinking agent, and by-products, such as cumyl alcohol and acetophenone, will be produced during the production process; even if it is removed by thermal degassing, it will cause structural damage to XLPE internally, increase transmission loss, and can even seriously affect the life of DC cable [6]. Therefore, research on modified polyethylene materials with high-temperature dielectric resistance is a top priority.

At present, the modification scheme of cross-linked polyethylene insulation materials mainly includes improving the cleanliness of insulation materials, nanoparticle modification, and chemical modification [7]. Nanoparticle modification is the introduction of nanofillers containing deep traps into the polymer; after nanomodification, polymer materials are improved in terms of inhibiting space charge accumulation, reducing current density, and enhancing DC breakdown strength [8,9], Although inorganic nanoparticles are typically used as nanofillers, their compatibility with polyolefin insulating materials is poor, they are difficult to disperse, it is simple to block the processing filter during actual production, and their development potential is constrained [10,11]. Chemical modification is the addition of functional organic small molecule compounds to polymers to improve their electrical resistance [12,13,14], mainly by two methods: physical blending and chemical grafting. However, the polar compatibility of organic small molecule compounds and polymers is very different, and it is easy for migration and accumulation to occur under long-term high-temperature and high field strength working conditions, which will affect the electrical resistance of the polymer. Therefore, chemical grafting of functional organic compounds is currently the focus of research. Grafting maleic anhydride (MAH) to improve the dielectric properties of polymers is currently a popular method. Quirke has theoretically investigated the energy distribution of traps generated by physical and chemical defects through first-principle electronic structure calculations, which sensibly show that polar groups can form carrier deep traps in polymers [15,16]. Lee demonstrates the successful chemical grafting of maleic anhydride (MAH) onto the macromolecular chain of low-density polyethylene (LDPE), showing that heteropolar space charge accumulation and current density in LDPE-g-MAH could be unambiguously suppressed [12]. But, because the boiling point of MAH is lower than the production temperature of XLPE cable, it is liable to evaporate and form bubbles at high temperature in the equipment pipeline, which not only affects the grafting efficiency, but also seriously reduces the dielectric properties of the material.

The current chemical graft modification technology can improve the dielectric properties of insulating materials, but it also has various limitations and shortcomings. Therefore, it is crucial to advance the technique for molecular grafting modification. Based on the mechanism of grafted molecules introducing traps, a new compound containing polar groups was introduced with bismaleimide ethane (BMIE). According to first-principle electronic structure calculations, BMIE, like MAH, can introduce deep traps inside the polymer, thus, having the potential to suppress space charge and improve dielectric properties. The boiling point of BMIE is much higher than the production and operating temperature of XLPE, avoiding the defects of decomposition and evaporation of MAH to form bubbles. At the same time, BMIE also has two double bonds, which can not only be grafted to the polymer molecular chain, but also play the role of an auxiliary crosslinking agent to improve the crosslinking efficiency of the polymer.

## 2. Materials and Methods

### 2.1. Material Preparation

In order to fully reflect the improved electrical resistance of cross-linked polyethylene by BMIE, this experiment uses unmodified cross-linked polyethylene as a blank control, and the materials used are as follows: LDPE (LD200GH, Sinopec Company Ltd., Beijing, China) as the basis material, dicumyl peroxide (DCP) as the cross-linking agent, bismaleimide ethane (BMIE, BirdoTech, Shanghai, China) as the grafting compound, and pentaerythritol ester antioxidant (Irganox1010).

The blends of the two materials are first prepared in a mixer. LDPE containing 1.8 wt% DCP and 0.3 wt% 1010 is agitated at 110 °C and 50 rpm for 5 min to obtain a homogeneous raw material for the preparation of XLPE; LDPE is mixed with 0.3 wt% 1010 and 0.3 wt% BMIE at 200 °C and 50 rpm for 15 min, and then 1.4 wt% DCP is included and stirred at 110 °C for 3 min after cooling to obtain raw materials for the uniform preparation of XLPE-g-BMIE [17,18].

**Figure 1:**
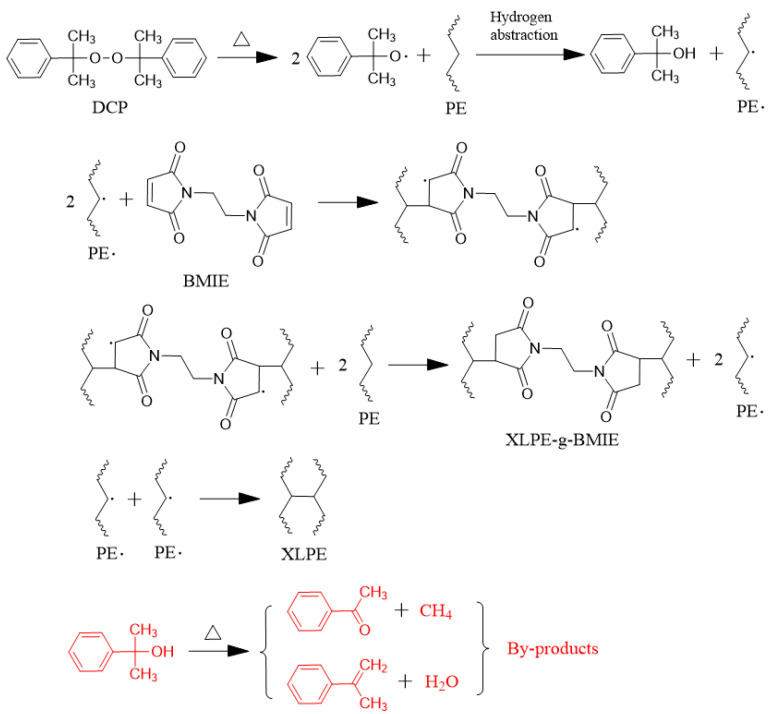
Reaction mechanism diagram of free radical-initiated BMIE grafting polyethylene and cross-linking.

The resulting blends are processed in a plate sulfurizer at 110 °C, where the pressure is increased by 5 MPa every 5 min from 0 to 15 MPa to melt the ingredients, followed by further processing at 175 °C for 30 min at 15 MPa. The peroxide bond in the DCP molecule is heated and homolytically cleaved to produce alkyl radicals. Alkyl radicals take hydrogen atoms from the polymer chains to produce peroxide decomposition products of kurkyl alcohols and polymer radicals. The radicals are transferred from the polymer to the BMIE molecule, causing the double bonds on the BMIE molecule to open and form carbon–carbon crosslinks with the polymer chains, and then the radicals further take over the hydrogen atoms of other polymer chains, eventually forming XLPE-g-BMIE and polymer radicals. A carbon–carbon crosslinking bond is also formed between the two polymer radicals, at which point the crosslinking reaction is completed. The peroxide decomposition product, kurkyl alcohol, is not stable and can be decomposed into acetophenone and methane or styrene and water at high temperatures, forming a series of decomposition by-products that negatively affect the electrical resistance of the material [19,20], then cooled to ambient temperature in a cooler at 15 MPa. The resulting material is thermally degassed in a vacuum oven at 80 °C for 48 h to relieve internal stress and remove reaction by-products to obtain the final product XLPE and the modified material XLPE-g-BMIE. The reaction mechanism is shown in Figure 1.

### 2.2. Characterization and Measurement

According to the standards of GB/T 2951.21-2008 [21] and ASTM D 2765-2011 [22], the thermal elongation and cross-linking degree of the specimen were tested through the thermal extension test and a gel extraction experiment. These methods are frequently used in power cable testing standards. To characterize BMIE molecules grafted onto polyethylene molecular chains, Fourier transform infrared (FT-IR) spectroscopy was used in the spectral range of 500~4000 cm^−1^ with a scanning resolution of 2 cm^−1^ to characterize the grafted BMIE in modified materials by IR transmission spectroscopy of 200 μm thick film samples. A pulsed electroacoustic system is used to measure the spatial charge distribution inside the material by applying an electric field of 40 kV/mm in polarization for 30 min and then shorting it for 30 min at an ambient temperature of 30–80 °C, in which the test material is made into a 50 × 50 × 0.3 mm^3^ sheet sample. Conductance currents are measured at temperatures from 20 to 80 °C by a three-electrode system for 100 mm diameter, 100 µm thickness sheet samples with aluminum electrodes on both sides. A ring-shaped protective electrode (inner and outer diameters of 54 mm and 76 mm, respectively) surrounds a disk of measuring electrodes (50 mm diameter) on one side of the membrane sample and a larger circular electrode of 78 mm diameter on the other side is used to apply the high voltage. At 20 °C, 40 °C, 60 °C, and 80 °C, a field strength of 5–50 kV/mm is applied to the test material for 30 min to measure the stable conductivity current, and the current density at different field strengths is calculated according to the ratio of the conductivity current to the specimen area. According to the international standard IEC 60243 [23], DC breakdown experiments are carried out using a DC breakdown system at a uniform ramp rate of 1 kV/s until the material with a thickness of 50 µm is broken down. To prevent surface discharge, the specimen and electrodes are immersed in silicone oil during the experiment. The voltage value at the time of breakdown is read, and the corresponding DC breakdown strength is calculated. The test results are processed using the Weibull statistical distribution method, and the breakdown field strength with a cumulative damage probability of 63.2% is taken as the characteristic breakdown field strength of the material.

### 2.3. Molecular Model and Theoretical Methodology

First-principle calculations [24,25] are employed to geometrically optimize the constructed initial polymer configurations for structural relaxation by total energy generalization minimization using a conjugate gradient algorithm. Electronic structure is calculated for molecular orbitals and the electronic density of states to study band edge features and trap states introduced by grafting [26,27]. First-principle calculations were performed by employing the Materials Studio 8.0 software package (Accelrys Inc., v8.0.0.843, San Diego, CA, USA). Detailed schemes and parameters used in the DMol3 calculations are shown in Table 1.

## 3. Results and Discussion

### 3.1. Testing and Characterization of Crosslinking

In order to verify whether adding BMIE and reducing the amount of the cross-linking agent DCP affects the degree of cross-linking of the polymer, thermal elongation tests and gel extraction experiments were used to test the relevant properties of the material in this paper, and the results are shown in Table 2.

From the data in the table, we can see that the thermal elongation and gel content of XLPE-g-BMIE and XLPE are basically the same, indicating that adding BMIE and reducing the amount of the cross-linking agent DCP do not affect the degree of cross-linking of the material. According to the standards of GB/T 2951.21-2008 and ASTM D 2765-2011, thermal elongation less than 80% is considered qualified, and gel content should be more than 80%. Combined with the data in Table 2, both XLPE-g-BMIE and XLPE meet the relevant standards.

### 3.2. Molecular Structure Characterization

**Figure 2:**
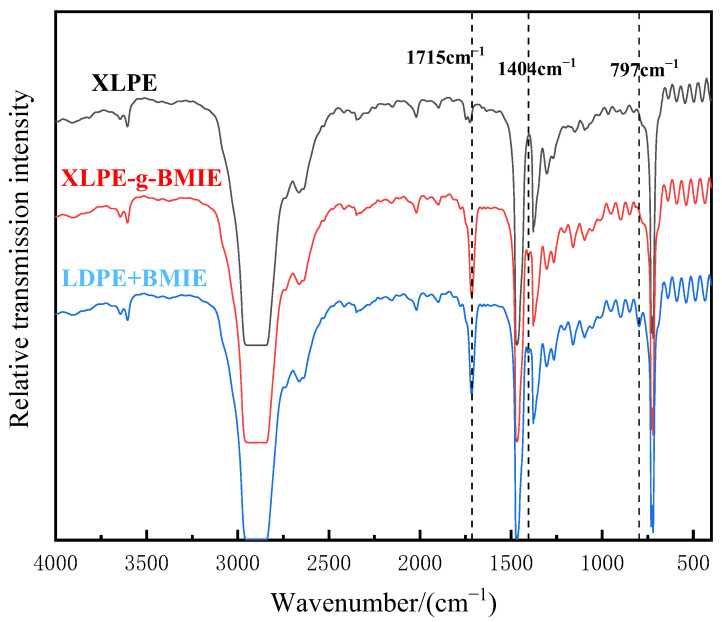
Infrared transmission spectra of XLPE, XLPE-g-BMIE, and LDPE + BMIE.

In order to determine whether BMIE was successfully grafted to the polyethylene molecular chain, Fourier transform infrared spectroscopy was used to chemically identify XLPE, XLPE-G-BMIE, and LDPE + BMIE. LDPE + BMIE is a physical blend of polyethylene and BMIE. The results are illustrated in Figure 2.

As can be seen in Figure 2, compared with XLPE, XLPE-g-BMIE has two new characteristic absorption peaks, namely the stretching vibration absorption peak of the carbonyl group (C=O) at 1715 cm^−1^ and the stretching vibration absorption peak of C-N bond at 1404 cm^−1^. The chemical structure of BMIE contains the above two functional groups. LDPE + BMIE has three new characteristic absorption peaks. In addition to the C=O bond and C-N bond absorption peaks, as in XLPE-g-BMIE, there is also a C=C bond stretching vibration absorption peak of cis-olefin at 797 cm^−1^. Since the material has undergone sufficient high-temperature degassing treatment before, there are basically no free small molecule compounds inside the material, and the IR results of XLPE-g-BMIE and LDPE + BMIE show that XLPE-g-BMIE has no C=C bond, so it can be judged that BMIE is successfully grafted to the polyethylene molecular chain by consuming the C=C bond.

### 3.3. Space Charge Characteristics

**Figure 3:**
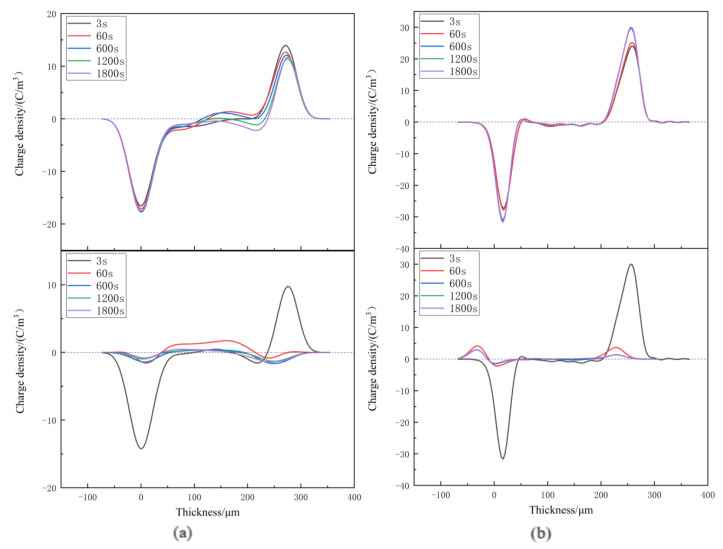
Space charge distributions at 30 °C in (**a**) XLPE and (**b**) XLPE-g-BMIE under applied DC electric field 40 kV/mm (upper panels) and in short-circuit (bottom panels).

**Figure 4:**
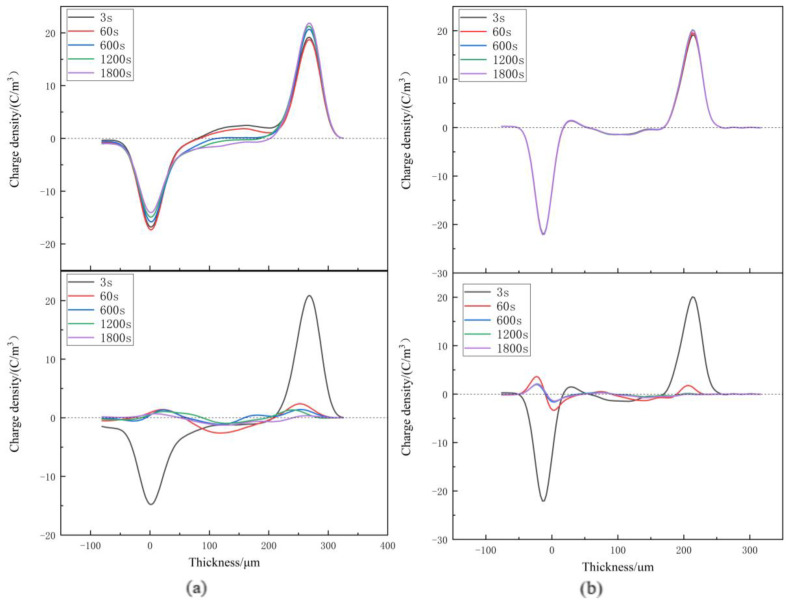
Space charge distributions at 40 °C in (**a**) XLPE and (**b**) XLPE-g-BMIE under applied DC electric field 40 kV/mm (upper panels) and in short-circuit (bottom panels).

**Figure 5:**
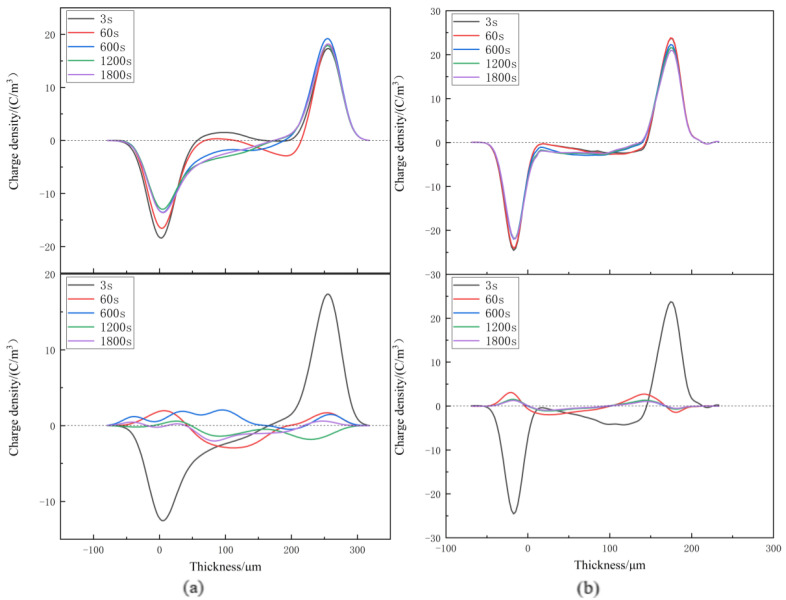
Space charge distributions at 60 °C in (**a**) XLPE and (**b**) XLPE-g-BMIE under applied DC electric field 40 kV/mm (upper panels) and in short-circuit (bottom panels).

**Figure 6:**
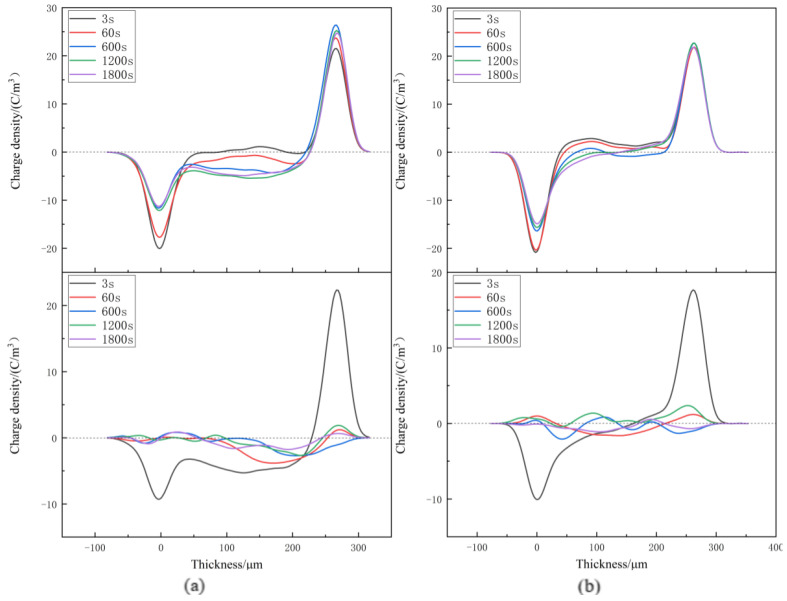
Space charge distributions at 80 °C in (**a**) XLPE, (**b**) XLPE-g-BMIE under applied DC electric field 40 kV/mm (upper panels) and in short-circuit (bottom panels).

Space charge distribution of XLPE and XLPE-g-BMIE at different temperatures from 30~80 °C was tested by the internationally popular pulsed electroacoustics (PEA) [29,30,31,32,33] to elucidate the space charge injection mechanism, and the results are shown in Figure 3, Figure 4, Figure 5 and Figure 6. The test results are divided into two parts: polarization and short-circuit. Between the two induced charge peaks is the space charge inside the material. During polarization and short-circuiting, the smaller the space charge density in the internal compartment of the material, the better the ability of the material to suppress space charge.

At 30 °C, during the polarization process, a small amount of homopolar charge accumulates near the negative electrode of XLPE, from the electrode injection. With the increase in time, the anisotropic charge begins to accumulate near the positive pole, which is caused by the decomposition of impurities within the material. With the increase in time, the charge density gradually increases, and the maximum accumulation is 2.23 C/m^3^. Compared with XLPE, there is no obvious space charge accumulation inside XLPE-g-BMIE, only a small amount of heteropolar space charge accumulation near the negative pole, which should be caused by the decomposition of impurities inside the material, and its maximum space charge accumulation is 1.01 C/m^3^. After the start of the short-circuit, the internal space charge of both materials starts to dissipate rapidly, and the rate of space charge dissipation tends to level off after 600 s for both. When the short-circuit is over, there is still a small amount of space charge left, and the average internal charge of XLPE is 0.57 C/m^3^, while the average internal charge of XLPE-g-BMIE material is 0.33 C/m^3^.

When the temperature rises to 40 °C, during the polarization process, the XLPE space charge accumulates significantly since 60 s, and the tendency of accumulating to the interior of the material is obvious, and the maximum accumulation of space charge inside the material is 2.47 C/m^3^. A small amount of anisotropic space charge accumulation near the negative pole of XLPE-g-BMIE should be caused by the decomposition of impurities within the material, and a small amount of space charge accumulation starts inside the material, and the maximum accumulation of space charge inside the material is 1.44 C/m^3^. During the short-circuit, the internal space charge of XLPE material dissipates slowly. When the short-circuit is 1800 s, the average internal charge of the material is 0.63 C/m^3^. The space charge inside the XLPE-g-BMIE material began to dissipate rapidly, and it was basically dissipated at 600 s. When the short-circuit is 1800 s, the average internal charge of the material is 0.42 C/m^3^.

At 60 °C, during the polarization process, the heteropolar charge around the two stages of XLPE was dominated by heteropolar charge, and later the homopolar charge was dominant, indicating that the source of space charge was rapidly changed from impurity decomposition to electrode injection. The space charge is injected into the material, obviously, and the maximum accumulation of space charge inside the material is 3.82 C/m^3^. With the increase in time, there is also a significant accumulation of homopolar space charge near the positive and negative electrodes of XLPE-g-BMIE, indicating that after the temperature rises, the accumulation rate of space charge in the material also accelerates, and the maximum accumulation of space charge inside the material is 2.90 C/m^3^. During the short-circuit, the internal space charge of XLPE material dissipates slowly, and the average internal charge of the material is 0.86 C/m^3^ at the end of the short-circuit. The average internal charge of XLPE-g-BMIE material is 0.55 C/m^3^.

At 80 °C, the space charge accumulation of XLPE material increases significantly during the polarization process, and the maximum space charge accumulation inside the material is 5.4 C/m^3^. With the increase in XLPE-g-BMIE, there is also obvious homopolar space charge accumulation near the positive and negative electrodes, the space charge accumulation rate further accelerates with the increase in temperature, and the maximum accumulation of space charge inside the material is 3.04 C/m^3^. During the short-circuit, a large amount of negative polar charge accumulates inside XLPE, and the location of the peak space charge gradually tends to be inside the inner material. After the end of the short-circuit, the average charge inside the material is 0.97 C/m^3^, and the average internal charge of XLPE-g-BMIE material is 0.55 C/m^3^.

In summary, we can conclude that the effect of suppressing the space charge of XLPE-g-BMIE is significantly improved compared to unmodified XLPE over the entire temperature range tested, as both the spatial charge accumulation during the polarization process and the space charge residual XLPE-g-BMIE after the short-circuit are much smaller than XLPE, indicating that the grafted BMIE can introduce carrier depth traps inside the polymer, thereby successfully inhibiting the accumulation of space charge inside the material.

### 3.4. Electric Current Density

The DC conductivity current of the specimen is tested at 20–80 °C with a three-electrode test system, the current density of the specimen is calculated, and the results are as follows.

**Figure 7:**
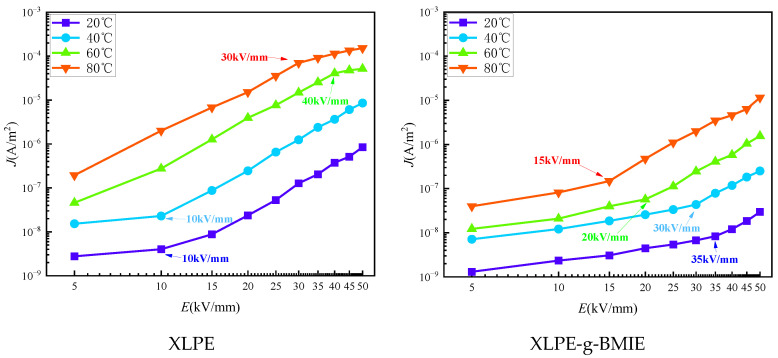
Conductivity properties of J–E varying curves for XLPE and XLPE-g-BMIE.

The J–E curves of current density versus field strength for the two materials at different temperatures are shown in Figure 7. From the figure, it can be seen that the current density of XLPE-g-BMIE is smaller than that of XLPE at different temperatures, showing excellent conductive current suppression. Meanwhile, the J–E curve of XLPE changes significantly with temperature, and its current density at the working field strength of the cable increases by nearly 650 times when the temperature rises from 20 °C to 80 °C, showing a strong temperature dependence, while the XLPE-g-BMIE under the same conditions only increases by about 100 times, showing a lower temperature dependence. In order to analyze the J–E curve more accurately, this paper uses the segmented linear fitting method to fit the J–E curve characteristics. Table 3 shows the current density slope (k_D_) and the threshold value of the electric field strength (E_Ω_) at the maximum curvature point at different stages.

As can be seen from Figure 7, at 20 °C and 40 °C, the J–E curves of both materials show a stage where k_D_ is approximately equal to 1 at low field strengths, and the charge conduction in the sample in this stage is dominated by ohmic conduction, so this region is also called the ohmic region (region I). As the field strength rises, the J–E curve of XLPE first appears E_Ω_ at 10 kV/mm, which marks the change in its conductive process from Ohm’s law to a sharply increasing nonlinear law, i.e., space charge-limited conductance (SCLC), and the J–E curve also enters the second stage. In the SCLC region (region II), the k_D_ of the J–E curve increases sharply to between 3–4, because the space charge begins to accumulate rapidly inside the material, and the rate of carriers in the material continues to increase, resulting in a nonlinear correlation of J–E in the SCLC region. At 60 °C, the ohmic zone of XLPE is no longer within the measurement range, and the J–E curve enters the SCLC region from the beginning of the measurement. With the increase in field strength, the J–E curve E_Ω_ appears again, which is 40 kV/mm, indicating that the carrier rate inside the insulator has reached the upper limit of material tolerance and cannot continue to increase, so its k_D_ also drops to between 1–2, which also marks the third stage (region III) of the J–E curve. At 80 °C, the J–E curve characteristics are similar to those at 60 °C, as the ohmic region is not within the measurement range, except that the E_Ω_ at the maximum curvature point of the second and third stages is 30 kV/mm, which is 10 kV/mm less than 60 °C. This indicates that as the temperature increases, the space charge accumulation rate becomes faster and the growth of the carrier rate within the material becomes larger, so the upper limit of the carrier rate within the material is reached more quickly and the J–E curve enters the third stage more quickly. After grafting BMIE and analyzing the data in Table 3, we find that the difference in k_D_ between XLPE-g-BMIE and XLPE is not significant, while E_Ω_ increases significantly in the same stage. The E_Ω_ of XLPE-g-BMIE is 35 kV/mm and 30 kV/mm at 20 °C and 40 °C, respectively, which is much larger than that of XLPE under the same experimental conditions, and the E_Ω_ of the J–E curve of XLPE-g-BMIE always exists in the ohmic region and does not enter the third stage in the measurement range, although it also decreases with the increase in temperature. The analysis shows that each transition in the conductive properties is closely related to the charge injection behavior of the electrode, which is modulated by the deep trap energy level introduced by the grafted BMIE. The molecular structure of BMIE contains polar groups which can introduce a large number of deep trap energy levels within the polymer, and these deep traps can capture the charge injected from the outer electrode and transported in the medium under the action of electric field. The trap charge located inside the medium near the electrode will form a charge dot matrix and generate a Coulomb shielding field, raising the electric field to inject charge potential barrier into the material. Only when the electric field varies in a wide range can the electrons obtain enough energy to cross the potential barrier, thus, inhibiting the accumulation of space charge, so its threshold field strength becomes larger and less dependent on temperature.

### 3.5. Electric Charge Traps

**Figure 8:**
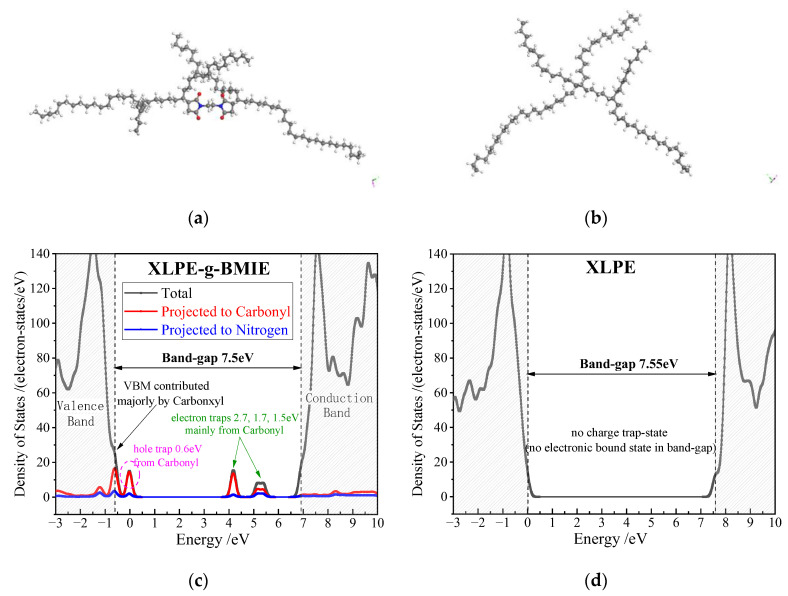
(**a**,**b**) Schematics of XLPE-g-BMIE and XLPE with gray, white, red, and blue spherules identifying carbon, hydrogen, oxygen, and nitrogen atoms, respectively; (**c**,**d**) density of states (DOS) of XLPE-g-BMIE and XLPE from first-principle calculations.

According to the first-principles method of the all-electron numerical orbital, the DMol3 program of the Materials Studio 8.0 software package is used to calculate the electron energy state density and atomic projection state density of XLPE-g-BMIE and XLPE, which is the theoretical basis for exploring whether grafted BMIE can effectively introduce deep trap energy levels. The results are shown in Figure 8.

Figure 8 shows the relaxed (geometrically optimized) model and state density (DOS) of XLPE-g-BMIE and XLPE. XLPE has no electron trapping energy levels in the energy band gap. The BMIE grafted onto the polyethylene produces three unoccupied electron-bound states and two hole-bound states in the polyethylene band gap, and the unoccupied electron-bound states farther from the bottom of the PE conduction band become deep traps for the conduction band electrons with deep trap energy levels of about 2.7 eV, 1.7 eV, and 1.5 eV. These deep trap energy levels are densely distributed in XLPE-g-BMIE, and when the electrode emits electrons into the dielectric at high fields, the electrons will enter the deep traps on the dielectric surface and form a uniformly distributed dense charge layer, which in turn forms a Coulomb force field and prevents further injection of electrons or holes on the electrode surface, thus, enhancing the material’s ability to suppress space charge and reducing the electrode’s carrier injection capability. In the meantime, the density of states near the band edge of the polymer molecule is involved in the probability of carrier conversion from adjacent electronic levels and, thus, dominates the carrier mobility without considering impurity or defect scattering. The grafted BMIE also introduces slightly lower energy level hole-bound states, one of which is very close to the valence band top (VBM) and, therefore, merges into the valence band to form a new VBM, leading to a significant decrease in DOS at the valence band edge, as shown in Figure 8c, which implies that a rather low carrier narrow shift rate is obtained and, therefore, consistently explains the reduced current density in the XLPE-g-BMIE. The projected density of states shows that the deep trap energy level at 2.7 eV is mainly contributed by the carbonyl group (>90%), with a small fraction coming from the nitrogen atoms in the BMIE (<10%), while the deep trap energy levels at 1.7 eV and 1.5 eV are 60% contributed by the carbonyl group and 40% by the nitrogen atoms, and the hole-bound energy level at 0.6 eV is basically contributed entirely by the carbonyl group. This also demonstrates that the polar group carbonyl can introduce electronic states in the band gap of polyethylene molecules with deep traps in energy levels, thus, effectively scattering and trapping electrons in the conduction band. The calculated results indicate that grafting BMIE can introduce a dense distribution of deep trap energy levels inside the polymer, resulting in shorter electron free range and reduced electron carriers, which is also consistent with the experimental results on electrical conductivity.

### 3.6. DC Breakdown Strength

**Figure 9:**
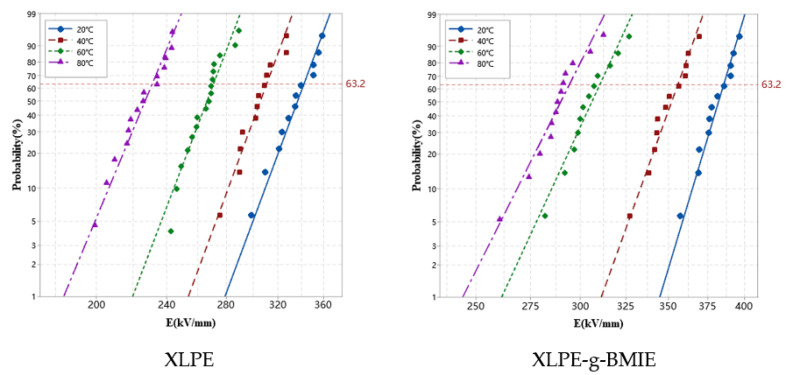
Dielectric breakdown strength (DBS) statistics fitted with Weibull distribution for XLPE and XLPE-g-BMIE at the temperature of 20–80 °C.

DC breakdown strength (DBS) tests were conducted on XLPE and XLPE-g-BMIE in the temperature range of 20–80 °C, and the measurements are shown in Figure 9. Table 4 shows the DC characteristic breakdown field strengths of the two materials at different temperatures. As can be seen from the table, the DBS of XLPE-g-BMIE is higher than that of XLPE at different temperatures, indicating that grafting BMIE significantly improves the DC breakdown characteristics of XLPE. With the increase in temperature, the DBS values of both samples decrease to different degrees. At 20 °C, 40 °C, 60 °C, and 80 °C, the DBS values of XLPE-g-BMIE are 12.6%, 14.3%, 14.5%, and 27.1% higher than XLPE, respectively, which means that even at high temperatures XLPE-g-BMIE still has good DC breakdown characteristics, and its electrical properties are less dependent on temperature.

Based on the experimental results and quantum mechanical calculations above, it is sensible that the enhancement of the DBS is associated with an increase in the trap energy level depth and density. The reason why grafting BMIE can improve the DBS of XLPE is that after grafting polar groups, a large number of densely arranged deep traps are formed inside the material, which can trap the injected charges when an electric field is applied, delaying the accumulation of charges in the internal space of the material, reducing the degree of electric field distortion inside the material, slowing down the local surge of electric field intensity due to electric field distortion, and, thus, improving the DBS of the material.

## 4. Conclusions

In this paper, bismaleimide ethane (BMIE) was grafted onto a polyethylene molecular chain by a free radical-initiated grafting technique to improve the DC dielectric properties of XLPE, and the modification mechanism was comprehensively investigated by combining electrical performance tests and first-principle calculations. The thermal elongation test and gel content test proved that XLPE-g-BMIE has a high crosslinking degree even with a 27.8% reduction in the amount of DCP used. The introduction of BMIE has two advantages. One is its high boiling point, unlike MAH, which has the problem of volatility; the other is that it reduces the amount of DCP, thus, reducing the impact of DCP decomposition by-products on the DC dielectric properties of the polymer. By analyzing the test results of breakdown field strength, conductivity current, and space charge distribution at 20–80 °C, it is proved that the deep trap introduced by grafted BMIE is the main reason for the improved DC dielectric performance of XLPE-g-BMIE. The charge trapped in the deep trap near the Coulomb potential shielding electrode can significantly hinder space charge injection, thus, reducing the conductivity current and the distortion field generated by space charge accumulation, which in turn increases the DC breakdown field strength. First-principle calculations also prove this. This study proposes a new solution for the grafting modification of DC high-voltage insulation materials, which is expected to be practically applied in the production of HVDC cables.

## Figures and Tables

**Table 1:** Schemes and parameters adopted in the first-principle calculations by DMol3.

Electronic Hamiltonian	Scheme	Condition and Parameter
Exchange correlation energy	Meta-generalized gradient approximation	M11-L [28]
Integration accuracy		2000 grid points/atom
	Tolerance	1 × 10^−6^ eV/atom
SCF	Multipolar expansion	Octupole
	Charge density mixing	Charge = 0.3, DIIS = 5
Core treatment	All-electron	
Numerical basis set	DNP	Basis file 4.4
Orbital cutoff	Global	5.0 Å

**Table 2:** Heat extension rate and gel content of two materials.

	Heat Extension Rate (%)	Gel Content (%)
XLPE	36	85.5
XLPE-g-BMIE	34	85.8

**Table 3:** The current density slope (k_D_) and threshold electrical field strength (E_Ω_) at different temperatures.

			XLPE	XLPE-g-BMIE
20 °C	k_D_ (A/(kVmm))	Ⅰ	0.84	0.94
		Ⅱ	3.83	3.53
		Ⅲ		
	E_Ω_ (kV/mm)		10	35
40 °C	k_D_ (A/(kVmm))	Ⅰ	0.88	1.00
		Ⅱ	3.75	3.42
		Ⅲ		
	E_Ω_ (kV/mm)		10	30
60 °C	k_D_ (A/(kVmm))	Ⅰ		1.11
		Ⅱ	3.33	3.56
		Ⅲ	1.01	
	E_Ω_		40	20
80 °C	k_D_ (A/(kVmm))	Ⅰ		1.18
		Ⅱ	3.23	3.46
		Ⅲ	1.52	
	E_Ω_ (kV/mm)		30	15

**Table 4:** The characteristic 63.2% DBS of Weibull distribution fitted in a 95% confidence interval at different temperatures (kV/mm).

	20 °C	40 °C	60 °C	80 °C
XLPE	342.1	311.3	271.1	231.6
XLPE-g-BMIE	385.4	355.7	310.3	294.1

## Data Availability

The data presented in this study are available on request from the corresponding author.
